# Knowledge, attitudes and health choices among non-diabetic patients regarding diabetes mellitus

**DOI:** 10.4102/jcmsa.v2i1.52

**Published:** 2024-03-29

**Authors:** Mbuyi Roland Tshibeya, Michele Torlutter

**Affiliations:** 1 Department of Family Medicine, Faculty of Health Science, University of the Witwatersrand, Johannesburg, South Africa

**Keywords:** diabetes mellitus, non-diabetic patients, knowledge, attitudes, health choices, primary healthcare

## Abstract

**Background:**

Healthcare workers require better understanding of the current knowledge and behaviours of the local community towards diabetes mellitus (DM) and its prevention before appropriate interventions can be developed to address the gaps. There is currently a paucity of scientific papers on knowledge, attitudes and health choices among the non-diabetic population in a facility setting in South Africa.

**Methods:**

In this cross-sectional study, a self-reported questionnaire was administered to 165 adult participants attending the Chiawelo Community Practice from 02 March 2020 to 17 April 2020. The study utilised descriptive statistics, Chi-square testing, univariate logistic regression, and multivariate analysis for variable assessment.

**Results:**

Almost half of the participants (49%) had good knowledge of DM, with 60% indicating good attitudes and 52% making good health choices to prevent DM. Participants having received education from clinicians were 5.61 times more likely to develop better attitudes and 3.92 times more likely to adopt better health choices towards DM compared to those who obtained information from media or other sources.

**Conclusion:**

The study found that poor knowledge of DM does not necessarily translate into poor attitude towards the disease, which is noteworthy. The study also highlighted the important role of healthcare workers in influencing behaviour change.

**Contribution:**

Information from this study can be used to strengthen health services through several clinical governance activities including quality improvement, capacity building, health education and community-oriented promotion, and prevention strategies.

## Introduction

The burden of disease from diabetes mellitus (DM) is ever-increasing. Currently, 1 in 11 adults is affected by DM globally, with 90% of those having type 2 DM.^1^ The prevalence of type 2 DM is projected to rise to 693 million by 2045, with half of the people affected remaining undiagnosed.^2^ The world spent approximately $850 billion on diabetic care in 2017.^2^ The global mortality rate from DM in 2019 was 11.3%.^3^

On the African continent, it has been shown that DM often goes undiagnosed. A systematic review in 2020 in this region revealed a prevalence of 3.85% (95% confidence interval [CI]: 3.10–4.60) of undiagnosed DM among adults.^4^ Compared to the rest of the world, Africa also has the highest percentage of deaths related to DM in individuals aged less than 60 years (73.1%).^3^

The prevalence of non-communicable diseases (NCDs) is reportedly increasing faster in poor and developing countries, and accounted for 57.4% of the total burden of disease in South Africa in 2016.^5,6^ A study published in South Africa in 2017 showed that the prevalence of DM in individuals older than 15 years was 10.1%, which demonstrated a slightly higher rate compared to the global statistics as stated above.^7^ According to Statistics South Africa, DM was the fifth most common cause of death in South Africa in 2012.^8^ South Africa is experiencing an increase in NCDs, including DM, because of several risk factors that may be modifiable or non-modifiable. The effect of urbanisation on populations as well as rural-urban differences have had a major impact on the increase in the risk for DM. Modifiable risk factors may include diminished physical exercises, diet, obesity and psychosocial factors. Of particular concern is the number of obese children, which in sub-Saharan Africa has increased to 10 million in 2012 from 4 million in 1990.^9^ Non-modifiable risk factors include family history, age and genetics.^10^ The focus of this research is mainly on interventions that address the modifiable risk factors of type 2 DM.

A study of 6958 patients in a Danish cohort, conducted in 2018, revealed that slightly more than one-third (35%) of type 2 DM patients had micro- and macrovascular complications related to DM at the time of diagnosis.^11^ During the latent phase of the disease, micro- and macrovascular complications can progress significantly. The risk of contracting diabetic neuropathy, which is the most common microvascular complication, and other microvascular complications, depends on the severity and duration of hyperglycaemia.^12^ Diabetic neuropathy can develop several years before type 2 DM is diagnosed.^12^ Once DM is established, the long-term complications may include cardiovascular disease (stroke and ischemic heart disease), renal failure, blindness and limb amputation.^13^ Diabetes mellitus also impairs the control of diseases such as human immunodeficiency virus (HIV) and tuberculosis.^14^ It is a condition that requires behaviour change in relation to the modifiable risk factors. If detected early, in the asymptomatic phases, the trajectory of the disease may be altered for better outcomes.^15^ This research was important to assess the awareness of the general population to the risks of DM, and the importance of lifestyle changes during the latent phase of illness when people do not yet know they have the disease.

Studies done in Asian and other African countries among patients diagnosed with DM have shown a significant relationship between good knowledge and attitudes towards diabetes and better blood glucose control.^16,17,18^ Health promotion in clinics and the community may therefore improve knowledge, attitudes and health choices in guarding against DM. Increased knowledge, however, does not necessarily result in a change of behaviour, and behaviour change is difficult for most people.^19^

Healthcare workers require better understanding of the current knowledge and behaviours of the local community towards DM and DM prevention before appropriate interventions can be developed to address gaps. Furthermore, the way in which information is delivered to patients must also be addressed. Clinical services are not always patient-centred, and health practitioners often have an authoritative style, which might not be conducive to conveying information to individuals with different background knowledge, beliefs, or specific ideas or needs.^20^

There is currently a paucity of scientific papers on knowledge, attitudes, and health choices among the non-diabetic population in a facility setting in South Africa. This study, which was conducted in the Chiawelo Community Practice (CCP), Soweto, South Africa, aimed to fill this gap. Approximately 1500 patients attend the CCP monthly. Of the total population registered in Ward 11 by the CCP’s community health workers, 1.17% reported being treated for DM at the clinic at the launch of this study, which may represent underdiagnosis of DM in this setting. Given the increasing prevalence, underdiagnosis and long pre-symptomatic phase in which prevention can be achieved, healthcare workers need to better understand the current lifestyle and health choices of patients. This information will assist in improving the behaviour and lifestyle choices in the community and better inform primary and secondary prevention measures. Information from this study can also be used to strengthen health services through several clinical governance activities, including quality improvement, capacity building, health education, and community-oriented promotion and prevention strategies.

## Methods

### Design and setting

This was a descriptive, cross-sectional study conducted at the CCP in a peri-urban region of Soweto, Johannesburg. The CCP is situated within the Chiawelo Community Health Centre, which is a government facility that serves mainly socioeconomically disadvantaged patients. It is a community-oriented primary healthcare facility run by family physicians, and provides a variety of services, including acute and chronic care, prevention and health promotion services, HIV management and family planning. Patients are seen by different health workers (health promoters, community health workers, nurses or doctors) in the chronic and acute areas of the facility, based on their reason for seeking medical advice or care.

### Study population, sample size and sampling

The study population comprised patients residing in Ward 11 who attended the CCP. While conducting this study, approximately 1500 patients were attending the CCP monthly. The study took place between March 2020 and April 2020 and included adult patients undiagnosed with type 2 DM, aged 18 years or older. Sample size was estimated using a single proportion sample size formula for prevalence studies, and was calculated as follows.

The following assumptions for random samples were considered: P = proportion at an 11.85% no questionnaire response, assuming a 95% CI Z = 1.96, D = margin of error (± 0.05) and adjusting for the design effect (DE = 1). Thus, the estimated sample size for this study was 161. With an attrition of 10%, the sample size was 177.^21^ Twelve participants were excluded because they had left blank spaces on the questionnaires; therefore, 165 participants were included in the analysis.

### Tools and data collection

The study used a structured, self-reported questionnaire designed by Kassahun and Mekonen. It was validated in Ethiopia but was adjusted to adapt it to the South African context, such as the currency from the Ethiopian birr to the South African rand.^22^ It was researcher-administered in the case of illiterate patients. The questionnaire was piloted with five patients randomly selected from the CCP and found to be suitable in terms of the appropriateness of language use, content, clarity, sequence and flow of the data collection tool. A research assistant, trained in the use of the research tool, assisted the primary researcher with obtaining informed consent as well as interpretation where a language barrier existed.

Every third patient in the queue was selected for the study. This occurred during routine weekday mornings while patients were waiting for their consultation in the acute and chronic areas of the CCP. The primary investigator explained the purpose of the study to the patients. Selected patients who agreed to participate were taken to a private room where the research assistant obtained their informed consent to participate in the study in their preferred languages. Patients able to read English completed the questionnaire themselves. It took 20 minutes on average to complete the questionnaire.

The data collection sheets were coded, transcribed to an Microsoft Excel spreadsheet under password protection, and stored on a flash disc kept safely in the researcher’s office. Confidentiality was strictly maintained, and only the primary researcher had access to the information.

### Analysis

Data transcribed in Microsoft Excel was uploaded to STATA version 15 (StataCorp LLC, College Station, Texas, United States [US]) for analysis. The normality of data distribution was determined using skewness, scatter plots, and kurtosis. For continuous variables, the median and standard deviations and interquartile ranges were utilised for normal and skewed data. For proportions and to assess differences within groups, the researcher used the Chi-square test or Fisher’s exact test. To check for associations, univariate logistic regression was done, and odds ratios were used to interpret the results. Multivariate analysis was done for variables with *p* < 0.25. An adjusted odds ratio (AOR) with *p* < 0.05 was regarded as statistically significant in this analysis. From the five-point Likert scale, the values were collapsed to two continuous data sets. The median was used to divide the categories.^23^ Kassahun and Mekonen defined health choices or practices as the usual participation of the community to prevent a chronic condition, and attitude as the manner in which a community thinks and acts towards a chronic condition.^22^ For attitude, the score range was 0–11 with a median of 7. Scores of 7 and above were regarded as good, and below 7 as poor. For health choices, the range was 0–5, with a median of 3. Scores of 3 and above were good and scores below 3 were poor. In this study, a range of scores were developed for knowledge. The range for knowledge was 0–31, with a median of 8. Scores below 8 were regarded as good, with scores 8 and above being regarded as poor knowledge.

### Ethical considerations

This research respected all ethical norms for research in medical fields involving human participants, preserving the ethical value of the World Medical Association’s Declaration of Helsinki. This study was not sponsored, and there was no conflict of interest, with autonomy to publish research results. Before conducting this study, a clearance certificate with registration number M190347 was obtained on 05 April 2019 from the Human Research Ethics Committee (HREC) (Medical) of the University of the Witwatersrand. Furthermore, the National Human Research Database and the Johannesburg District Research Committee also granted authorisation to conduct the study at the CCP on 14 January 2020, with project registration numbers GP201905007 and 2019-05-004, respectively. Furthermore, written consent was obtained from the participants of the study. All the above documents are available on request.

## Results

The study found that poor knowledge of DM does not necessarily translate into poor attitude towards the disease. Almost half of participants, 81 (49%), had good knowledge of DM, with 99 (60%) indicating good attitudes and 86 (52%) making good health choices to prevent DM.

### Socio-demographics

Table 1 illustrates the socio-demographics of the study population. The study had a total of 165 participants, with 112 (68%) being female. There was a good mix of age ranges in the study, with an anticipated large number of patients aged over 45 years (*n* = 57, 35%). The majority of participants were literate (*n* = 161, 98%), single (*n* = 103, 62%), and in a low income range (*n* = 126, 76%). Most had previously been exposed to DM education (*n* = 139, 84%), with the main sources being media (*n* = 60, 36%) and health practitioners (*n* = 49, 30%). Over half had a positive family history of DM (*n* = 99, 60%).

**TABLE 1:** Socio-demographic profiles of participants to evaluate knowledge, attitudes, health choices and related factors towards diabetes mellitus among members attending the Chiawelo community practice, Soweto, 2020 (*N* = 165).

Variable categories	*n*	Proportion %
**Gender**		
Male	53	32
Female	112	68
**Age category**		
≤ 24	27	16
25–34	42	25
35–44	39	24
45+	57	35
**Education**		
Illiterate	3	2
Literate	162	98
**Marital status**		
Single	103	62
Married	34	21
Other	28	17
**Occupation**		
Housewife	28	17
Student	25	15
Government/private	29	18
Daily labourer	23	14
Other	60	36
**Average monthly income**		
R0.00 – R4999.00	126	76
R5000.00 – R9999.00	39	24
**Exposure to DM education**		
Yes	139	84
No	26	16
**Information sources**		
Media	60	36
Health practitioners	49	30
Friends/relatives	28	17
Other	28	17
**Family history of DM**		
Yes	99	60
No	66	40

DM, diabetes mellitus.

### Knowledge

Questions related to knowledge were answered by 165 participants. Table 2 illustrates the responses to these questions on the definition, symptoms and signs, risk factors, complications and prevention of DM. Most participants were able to define DM correctly. Ninety-nine (60%) knew DM was a disease of inadequate production of insulin, 115 (70%) knew DM was not curable, and 99 (60%) knew DM could affect any part of the body. Only 80 (48%) participants knew about the more detailed relationship between insulin and DM.

**TABLE 2:** Frequency distribution of participant responses to diabetes mellitus knowledge, 2020 (*N* = 165).

Knowledge categories	Yes		No	
	*n*	%	*n*	%
**Definition of DM**				
DM is a disease of inadequate production of insulin	99	60	66	40
DM is a disease of the body not reacting to insulin	80	48	85	52
DM is not curable	115	70	50	30
DM is a disease that can affect any part of the body	99	60	66	40
**Signs and symptoms of DM**				
Frequent urination	117	71	48	29
Excessive thirst	122	74	43	26
Excessive hunger	121	73	44	27
Weight loss	112	68	53	32
High blood sugar level	111	67	54	33
Blurred vision	129	78	36	22
Slow healing of wounds	123	75	42	25
Feeling of weakness	111	67	54	33
**Risk factors of DM**				
Older age	64	39	101	61
Hereditary or family history of DM	100	61	65	39
Overweight or obesity	105	64	60	36
Pregnancy	64	39	101	61
Poor dietary habits	89	54	76	46
Lack of exercise and sedentary life	94	57	71	43
**Complications of DM**				
Eye problems or blindness	130	79	35	21
Kidney failure	107	65	58	35
Heart failure	93	56	72	44
Stroke	113	68	52	32
Amputation of limb	117	71	48	29
**Prevention of complications related to DM**				
Insulin injection	131	79	34	21
Tablets and capsules	126	77	39	23
Regular exercises	131	79	34	21
Healthy diet practice	140	85	25	15
Routine eye check and care	121	73	44	270
Feet and toes medical check up	95	57	70	43
Weight reduction	103	62	62	38

DM, diabetes mellitus.

### Attitudes and health choices

Table 3 illustrates participant responses to the questions assessing their attitudes towards DM, as well as options related to health choices that mitigate DM. Most participants tended to agree with the statements on screening, examination, disclosure to family, supportive relationships and sugar and weight management. However, the responses related to the effect of DM on marriage, daily activities and the management of certain lifestyle factors such as smoking and exercising tended to be more ambiguous as shown in Table 3.

**TABLE 3:** Frequency distribution of patient responses to attitudes towards diabetes mellitus and self-reported health choices that prevent or mitigate diabetes mellitus, 2020 (*N* = 165).

Attitude towards DM questions	Agree		Disagree		Frequent		Less frequent	
	*n*	%	*n*	%	*n*	%	*n*	%
Would you mind disclosing your DM status?	130	79	35	21	-	-	-	-
Do you think you should be examined for DM?	148	90	17	10	-	-	-	-
Do you think family members should be screened for DM?	128	77	37	23	-	-	-	-
Is support from friends and family advisable in addressing DM?	142	86	23	14	-	-	-	-
Should we avoid consumption of excessive sugar to control DM?	112	68	53	32	-	-	-	-
DM does not seriously affect marriage	60	36	105	64	-	-	-	-
DM does not affect daily activities	54	32	111	68	-	-	-	-
Physical exercises can prevent DM	79	38	86	62	-	-	-	-
Do you discuss smoking cessation with your clinician?	70	42	95	58	-	-	-	-
Maintaining a healthy weight helps in the management of DM	125	76	40	24	-	-	-	-
A well-controlled blood glucose level can prevent DM complications	123	75	42	25	-	-	-	-
**Health choices questions**								
How often do you consume fatty food	-	-	-	-	48	29	117	71
How often do you exercise 30–60 min	-	-	-	-	90	54	75	46
How often do you try maintaining your weight	-	-	-	-	85	51	80	49
How often do you smoke and drink alcohol	-	-	-	-	26	15	139	85
How often do you regularly screen for your blood sugar	-	-	-	-	60	37	105	63

DM, diabetes mellitus.

### Univariate and multivariate logistic regression

Univariate and multivariate logistic regression related to knowledge, attitudes and health choices levels are illustrated in Table 4, Table 5 and Table 6, respectively.

**TABLE 4:** Univariate and multivariate logistic regression predicting diabetes mellitus-associated knowledge among members attending the Chiawelo community practice, Soweto, 2020 (*N* = 165).

Variable category	Knowledge level				COR	95% CI	*p*	AOR	95% CI	*p*
	Not knowledgeable		Knowledgeable							
	*n*	%	*n*	%						
**Gender**										
Male	25	30	28	35	0.8	0.42–1.54	0.509	-	-	-
Female (Ref)	59	70	53	65	-	-	-	-	-	-
**Age (years)**								-	-	-
24 and below	14	17	13	16	1.28	0.51–3.21	0.593	-	-	-
25–34	22	26	20	25	1.31	0.59–2.92	0.506	-	-	-
35–44	22	26	17	21	1.54	0.68–3.50	0.300	-	-	-
45 and above	26	31	31	38	-	-	-	-	-	-
**Marital status**								-	-	-
Single (Ref)	48	73	55	56	-	-	-	-	-	-
Married	10	15	24	24	2.09	0.91–4.82	0.082	1.87	0.96–5.11	0.217
Other	8	12	20	20	2.18	0.88–5.40	0.092	2.08	0.74–5.80	0.160
**Level of education**										
Unable to read and write	3	4	0	0	-	-	-	-	-	-
Able to read and write	81	96	81	100	-	-	-	-	-	-
**Occupation**										
Housewife	10	15	18	18	0.97	0.38–2.48	0.948	1.02	0.35–2.98	0.977
Student	11	17	14	14	0.69	0.26–1.77	0.436	0.84	0.28–2.51	0.762
Government or private employer	12	18	17	17	0.76	0.31–1.89	0.560	1.12	0.40–3.17	0.818
Daily labourer	12	18	11	11	0.49	0.18–1.31	0.156	0.57	0.19–1.77	0.335
Other (Ref)	21	32	39	40	-	-	-	-	-	-
**Average family income**										
R0.00 – R4999.00 (Ref)	54	82	72	73	-	-	-	-	-	-
+R5000.00	12	18	27	27	1.69	0.78–3.63	0.181	1.53	0.64–3.66	0.335
**Family history of DM**										
Yes (Ref)	34	40	65	80	-	-	-	-	-	-
No	50	60	16	20	5.97	2.97–12.02	< 0.001	3.69	1.75–7.78	0.001
**Exposure to DM health education**										
Yes (Ref)	70	83	69	85	1.15	0.50–2.66	0.744	-	-	-
No	14	17	12	15	-	-	-	-	-	-
**Information source**										
Media	36	13	24	30	-	-	-	-	-	-
Health practitioners	19	22	30	37	0.42	0.19–0.91	0.029	2.08	0.85–5.09	0.108
Friends or relatives	9	11	19	23	0.32	0.12–0.81	0.017	2.16	0.75–6.28	0.155
Other	20	24	8	10	1.67	0.63–4.39	0.302	0.56	0.21–1.51	0.251

COR, crude odds ratio; AOR, adjusted odds ratio; CI, confidence interval.

**TABLE 5:** Univariate and multivariate logistic regression predicting DM-associated attitudes among members attending the Chiawelo community practice, Soweto, 2020 (*N* = 165).

Variable category	Attitudes level				COR	95% CI	*p*	AOR	95% CI	*p*
	Poor		Good							
	*n*	%	*n*	%						
**Gender**										
Male (Ref)	27	41	26	26	0.51	0.27–1.00	0.05	1.45	0.69–3.03	0.323
Female	39	59	73	74	-	-	-	-	-	-
**Age (years)**										
24 and below (Ref)	11	17	16	17	-	-	-	-	-	-
25–34	16	24	26	26	0.79	0.31–2.01	0.616	2.29	0.78–6.74	0.132
35–44	19	29	20	20	0.88	0.38–2.00	0.759	1.27	0.41–3.89	0.678
45 and above	20	30	37	37	0.57	0.25–1.31	0.183	2.37	0.85–6.60	0.098
**Marital status**										
Single (Ref)	52	62	51	63	-	-	-	-	-	-
Married	18	21	16	20	0.91	0.42–1.96	0.804	-	-	-
Other	14	17	14	17	1.02	0.44–2.35	0.964	-	-	-
**Level of education**										
Unable to read and write	2	3	1	1	0.33	0.03–3.68	0.365	-	-	-
Able to read and write	64	97	98	99	-	-	-	-	-	-
**Occupation**										
Housewife (Ref)	12	14	16	20	1.43	0.58–3.52	0.442	-	-	-
Farmer	14	17	11	13	0.84	0.33–2.15	0.715	-	-	-
Government/Private employer	14	17	15	19	1.15	0.47–2.78	0.764	-	-	-
Daily labourer	13	15	10	12	0.82	0.31–2.16	0.692	-	-	-
Other	31	37	29	36	-	-	-	-	-	-
**Average family income**										
R0.00 – R4999.00 (Ref)	63	75	63	78	-	-	-	-	-	-
+R5000.00	21	25	18	22	0.86	0.42–1.76	0.675	-	-	-
**Family history of DM**										
Yes (Ref)	31	47	68	69	-	-	-	-	-	-
No	35	53	31	31	0.4	0.21–0.77	0.006	0.46	0.23–0.94	0.034
**Exposure to DM health education**										
Yes (Ref)	54	82	85	56	-	-	-	-	-	-
No	12	18	14	14	0.74	0.32–1.72	0.486	-	-	-
**Information source**										
Media	29	44	31	31	-	-	-	-	-	-
Health practitioners	7	11	42	43	5.61	2.18–14.5	< 0.001	3.61	1.51–8.64	0.004
Friends or relatives	16	24	12	12	0.7	0.28–1.73	0.442	0.9	0.34–2.38	0.832
Other	14	21	14	14	0.94	0.38–2.29	0.884	1.1	0.42–2.9	0.849

DM, diabetes mellitus; COR, crude odds ratio; AOR, adjusted odds ratio; CI, confidence interval.

**TABLE 6:** Univariate and multivariate logistic regression predicting diabetes mellitus-associated health choices among members attending the Chiawelo community practice, Soweto, 2020 (*N* = 165).

Variable category	Health choices level				COR	95% CI	*p*	AOR	95% CI	*p*
	Poor choices		Good choices							
	*n*	%	*n*	%						
**Gender**										
Male (Ref)	27	34	26	30	0.83	0.43–1.61	0.588	-	-	-
Female	52	66	60	70	-	-	-	-	-	-
**Age (years)**										
24 and below	10	13	17	20	1.53	0.60–3.91	0.374	-	-	-
25–34	23	29	19	22	0.74	0.33–1.65	0.468	-	-	-
35–44	19	24	20	23	0.95	0.42–2.14	0.897	-	-	-
45 and above	27	34	30	35	-	-	-	-	-	-
**Marital status**										
Single (Ref)	47	59	56	65	-	-	-	-	-	-
Married	14	18	20	23	1.20	0.55–2.63	0.651	-	-	-
Other	18	23	10	12	0.47	0.20–1.11	0.084	-	-	-
**Level of education**										
Unable to read and write	2	3	1	1	0.45	0.04–5.09	0.521	-	-	-
Able to read and write	77	97	85	99	-	-	-	-	-	-
**Occupation**										
Housewife (Ref)	14	18	14	16	1.14	0.47–2.80	0.771	1.33	0.50–3.50	0.566
Student	9	11	16	19	2.03	0.78–5.31	0.148	2.34	0.83–6.58	0.106
Government or private employer	12	15	17	20	1.62	0.66–3.97	0.292	2.14	0.81–5.72	0.126
Daily labourer	12	15	11	13	1.05	0.40–2.74	0.925	1.3	0.46–3.65	0.622
Other	32	41	28	32	-	-	-	-	-	-
**Average family income**										
R0.00 – R4999.00 (Ref)	64	81	62	72	-	-	-	-	-	-
+R5000.00	15	19	24	28	1.65	0.79–3.44	0.180	1.51	0.69–3.36	0.301
**Family history of DM**										
Yes (Ref)	39	49	60	70	-	-	-	-	-	-
No	40	51	26	30	0.42	0.22–0.80	0.008	0.54	0.27–1.10	0.089
**Exposure to DM health education**										
Yes (Ref)	66	84	73	85	-	-	-	-	-	-
No	13	16	13	15	0.90	0.39–2.09	0.814	-	-	-
**Information source**										
Media	38	48	22	25		-	-	-	-	-
Health practitioners	15	19	34	40	3.92	1.75–8.74	0.001	3.60	1.54–8.39	0.003
Friends or relatives	11	14	17	20	2.67	1.06–6.71	0.037	2.65	1.01–6.99	0.048
Other	15	19	13	15	1.50	0.60–3.71	0.385	1.48	0.57–3.894	0.420

DM, diabetes mellitus; COR, crude odds ratio; AOR, adjusted odds ratio; CI, confidence interval.

Table 4 reflects that 81 (49%) participants had knowledge of DM. All 81 (100%) participants who were able to read and write were knowledgeable about DM. More than half of the participants (*n* = 57, 71%) who came from the income category R0.00 – R4999.00 knew about DM. Participants with no family history of DM were 3.69 times more likely to have poor knowledge of DM compared to those who had a family history.

Table 5 demonstrates that individuals with no family history of DM were 0.46 times less likely to have good attitudes towards DM compared to those with a family history of DM. Those who obtained their information from health practitioners were 3.61 times more likely to have good attitudes towards DM in comparison to those whose information came from media.

Table 6 reflects that participants who received their information from health practitioners were 3.6 times more likely to have good health choices compared to media as information source.

## Discussion

This study aimed to understand the knowledge, attitudes and behaviour of a peri-urban segment of the population, living under poor socio-economic circumstances. We wanted to understand the spread of ideas and lifestyle choices across different age groups among those undiagnosed with DM, thereby providing an opportunity for early intervention.

The first important consideration was the demographics of the study population. Sixty-eight per cent of participants were women; consequently, the study was skewed towards the knowledge, attitudes and behaviours of women. This may be important because it has been found that women generally have better help-seeking behaviour and possibly better health choices than men,^24^ although this was not investigated in this study. Conversely, women are generally more socio-economically disadvantaged than men, which may affect their educational attainment and ability to carry out desired health behaviours.^24^ Compared to the clinic population, the differences between the health behaviours of men and women might be even greater in the general community, which would need further research.

The gender distribution of those attending clinics is not unique to this study. A study on diabetes-related knowledge, attitudes and health choices among adult type 2 DM patients attending community healthcare centres in the Free State province in South Africa showed that female patients (76.1%) attended clinics more than men (23.9%).^25^ Men generally present late and have different help-seeking behaviour.^24^ A study conducted in Trinidad on the role of gender on attendance and compliance at an outpatient clinic for type 2 DM, found that more women (74.2%) than men (25.8%) attended the clinic and that they had better compliance to treatment than men.^26^ Men therefore become a special group that may be difficult to access, requiring a different approach to health promotion, lifestyle intervention and prevention and early detection of DM. The same applies to younger people who are harder to access from the clinic base. Attention also needs to be paid to children and obesity, where a family-oriented approach becomes important.

In general, participants knew about the signs and symptoms of DM: 117 participants (71%) knew about frequent urination, 122 (74%) about excessive thirst, and 121 (73%) about excessive hunger. Furthermore, 112 participants (68%) were aware of weight loss, 111 (67%) knew about high blood sugar, 129 (78%) about blurred vision, and 123 (75%) about slow wound healing. Participants, however, did not perform well on some questions related to risk factors for DM. Only 64 participants (39%) knew about older age and gestational diabetes (DM that occurs in pregnancy) as risk factors, with slightly more than half (*n* = 89, 54%), knowing that poor dietary habits and a sedentary life increased the risk. However, 100 participants (61%) were aware that family history was a risk factor, with 105 (64%) knowing that obesity or being overweight increased the risk.

The majority of participants were aware of DM complications, with 130 (79%) knowing about the eyes being affected, 107 (65%) about the kidneys, 93 (56%) about the heart, 113 (68%) about the brain, and 117 (71%) about the limbs. Most participants also knew how to treat DM: 131 (79%) knew insulin injections were used to treat DM, and 126 (76%) knew about the use of tablets and capsules. In terms of lifestyle management, 131 (79%) knew that regular exercise improved DM, 140 (85%) knew that a healthy diet was conducive to the treatment of DM, and 103 (62%) knew that weight reduction assisted in the prevention and management of the disease. In terms of the monitoring and prevention of DM complications, 121 (73%) participants knew of the eye test requirement, and 95 (57%) were aware of the necessity for foot care.

Regarding the questions on health choices, 139 (85%) participants reported that they smoked, or that they drank alcohol less frequently or not at all. Twenty-nine per cent of participants reported that they consumed fatty foods frequently or very frequently; 83 (51%) were conscious of maintaining their weight in the frequent and very frequent ranges; and 75 (46%) participants exercised less frequently or not at all. In terms of screening for DM, presumably in the clinic setting as these participants were non-diabetic, 61 (37%) reported frequent or very frequent blood sugar checks.

A need for intervention with reference to improved knowledge and change in behaviour was identified in this study, while recognising the complex relationship between knowledge and behaviour change. Studies have shown differing results when considering this complexity. Abbasi et al. conducted a knowledge, attitudes and health choices study in 2018 on Type 2 DM patients in the Kuala Muda district of Malaysia, and revealed that good knowledge led to better attitudes and health choices.^27^ This was also found by Kassahun and Mekonen in 2017.^22^

Other studies on diabetic knowledge, attitudes and health choices have shown that poor knowledge correlated with poor attitudes and health choices.^25, 28, 29, 30^ Conversely, it was found that good knowledge correlated with poor attitudes and health choices.^19^ A South African study conducted in 2010 confirmed these findings, showing that even though participants were educated on hypertension, which is also a chronic condition, adequate knowledge did not necessarily result in behavioural change.^31^ In this study, however, when considering the relationship between DM knowledge, attitudes and health choices, it was found that poor knowledge did not translate into poor attitudes as even participants who were not knowledgeable about DM had good attitudes and were trying to lead a healthier lifestyle in the absence of diabetes.

Sixty per cent of participants in this study had a positive family history of DM, demonstrating the prevalence and burden of DM and NCDs in this community. Of those, 65 (80%) were knowledgeable about the disease, whereas participants without a family history of DM were 5.97 times less knowledgeable, which was statistically significant (*p* < 0.001). This demonstrates that for most participants a family history translated into better knowledge. It was also shown that those without a family history for DM performed 0.46 times lower on attitudes than those with a family history of DM (*p* = 0.034). Nevertheless, attitudes to lifestyle management can be improved in those with DM in the family. It is important for healthcare workers to take note of this, and it may be important to target and treat not only diabetic patients but also their family units. Because of the strong genetic links of the disease, it could be a case of finding an early lifestyle intervention in such families. A family history of DM is often a missed risk factor in consultations and may link with undiagnosed DM and impaired fasting glucose information.^32, 33, 34^ This is an area where targeted prevention strategies and effective screening programmes may improve outcomes.

A study conducted in Ethiopia in 2017 confirmed our findings that participants without diabetes in the family had less knowledge than those who had diabetes in their family.^35^ In their study, knowledge was found to be 2.94 times (95% CI: 1.87–4.86) higher for those who had DM in the family. They also found the probability of having good health choices to be 3.38 times (CI: 2.05–5.58) higher compared to participants who did not have a positive family history. These findings underscore that health systems and delivery can be strengthened by focussing on families with diabetic members, where the risk is higher.

In this study, most participants were single (62%), and found to be 2.09 times more likely to have poor knowledge of DM compared to those who were married (*p* = 0.082). Marriage has been reported as a positive contributor to health-related behaviours and compliance with treatment.^36^ Involvement of spouses in their partners’ disease management improves stress management and the everyday quality of interactions between the couple.^37^ This information should be carefully sought in consultations using a holistic technique, and patients outside marriage should especially be targeted for improving lifestyle habits, which should also be prioritised within the community. We could not demonstrate a statistically significant association between DM and knowledge, attitudes and health choices, possibly because this study may not have been sufficiently representative.

Our study was different in that it looked at the non-diabetic population. It illuminated the gap in DM knowledge, with less than half (49%) of the participants being knowledgeable, which needs addressing. This study also showed that health workers were more effective than other sources of information at educating patients, as participants who received education from health workers were 5.61 times more likely to develop better attitudes to monitoring, prevention and behaviour change compared to those who obtained information from media (*p* < 0.001). Those participants were also 3.92 times more likely to adopt better health choices compared to those who obtained information from media and other sources (*p* = 0.001).

This leads to a further discussion on the importance of different information sources to educate the community and how they compare, and if they actually result in behaviour change. This study highlighted the fact that participants obtained medical knowledge from different sources. Media was the most common source of information (36%), followed by health practitioners (30%) and friends or relatives (17%). Media is therefore an important source of information, which should be utilised to a greater extent to ensure that correct messaging is achieved. It should be kept in mind that inaccurate health news can cause harm to health and tarnish the reliability of healthcare providers and medicine.^38^

In this study, it was found that only 49 (30%) participants received information or health education about diabetes from healthcare workers. A similar finding was observed in a community in southern Nigeria, where 27.9% of participants had health practitioners as their source of information.^39^ Patient-friendly delivery of information that is facilitative rather than directive needs implementing and may take place as health messaging to groups of patients by medical staff, or one-on-one in the consulting room by clinicians. It appears that a personalised touch is more effective than media at resulting in behaviour change, which is another area for exploration. Improved interviewing skills and brief motivational counselling skills by clinicians should be harnessed during consultations, as well as community-based interventions that have a more personalised touch and are more likely to translate into behaviour change.

Other findings in this research indicated that a large proportion of patients experienced ambivalence towards managing important lifestyle risk factors. This was demonstrated in the attitudes section where attitudes towards the effect of chronic diseases on marriage and relationships, effects on daily activities, and choices around exercise and smoking had a larger range of responses. This is an area where targeted prevention strategies may assist in the non-diabetic population to avert disease. In this study, 38% of participants admitted smoking and drinking alcohol. A study involving 732 participants in Limpopo, South Africa, however, revealed that only 13.7% were smokers and only 16.3% consumed alcohol.^40^ These differing findings were confirmed by the results of a study conducted in urban Pretoria and rural Bela Bela in South Africa which showed that the prevalence of both smoking and alcohol was twice as frequent among urban participants compared to rural participants.^41^ They also found that tobacco use was highly associated with the use of alcohol. Based on the above findings, targeting these lifestyle risk factors is therefore an important component in the prevention and management of most NCDs, especially in urban areas.

The current study found that only 52% of patients undiagnosed with DM were practising good health choices. This is of public health concern, given the increasing incidence of NCDs and the consequent implications. This study revealed that 15% of participants were in the range of harmful drinking and smoking, and 29% reported high fat diets, which may be an indicator of poor dietary choices. Not only should media be better utilised to spread information and address behaviour change, but health practitioners should also be trained in the facilitatory techniques of brief behaviour change counselling.^42^ This should be done in health facilities and also extend into communities. Changing behaviour requires a paradigm shift in the way we live and needs careful consideration as we see the impact urbanisation has on health. It is an important target in primary care facilities to promote health and assist people in making healthy choices. This might be challenging, as individuals might not feel in control of their personal circumstances and their environment. Healthcare providers, however, have to be able to assist patients in moving towards empowerment.^43^

### Study limitations

The study sample was selected from a facility-based population, which may not be representative of the community at large as it did not include those with pre-diabetes who were not utilising the health facility and were therefore presumed healthy. The sample size was small, even though a web-based statistical and epidemiologic calculator for public health was used. Also, the study was conducted in a brief timeframe as a cross-sectional survey during the coronavirus disease 2019 (COVID-19) period and may not describe the whole picture of this peri-urban community that has more than 3000 households. Women were over-represented in the study, with men generally having poorer help-seeking behaviour and possibly different knowledge, attitudes and health choices. The younger population, who may have riskier lifestyle attitudes and behaviours, were also underrepresented. We could also not demonstrate in this study the relationship between food insecurity and attitudes to health or lifestyle choices. The fact that the median was used may constitute a weakness or limitation in terms of measuring good and poor knowledge, attitudes or health choices.

The study may be biased because of the nature of the study tool that gave participants a tick sheet of options on diabetic knowledge, which may not be a true representation of knowledge. This may have given a false impression of the participants’ knowledge, where lack of understanding of questions, fear, embarrassment or dishonesty in reporting accurately may have played a role. Culture and tradition may also apply to the aforementioned considerations. Furthermore, it is always advisable to run the Cronbach’s Alpha test after the data collection to support a good internal consistency of the study instrument in the local population, which we did not do. The study had a power of 80 in the sample size, strengthening the analysis adjustment, thereby catering for other biases such as type 1 and type 2 errors.^44^

## Conclusion

From the findings of this study, we concluded that information sources and a family history of DM influenced knowledge, attitudes and health choices. The findings also highlighted the important role of healthcare workers in influencing behaviour change. Contrary to previous studies, this study illustrated that poor knowledge of DM did not necessarily translate into poor attitudes in preventing chronic conditions.

The study was useful because it highlighted the gaps where targeted prevention strategies are required to prevent DM and NCDs. This should be tailored to the different groups in the population studied by using media optimally, through health promotion by healthcare workers, and by community-based interventions.
